# Nursing Students’ Health Literacy and Strategies to Foster Patients’ Health Literacy

**DOI:** 10.3390/ijerph21081048

**Published:** 2024-08-09

**Authors:** Veronika Anselmann, Simone Halder, Sophie Sauer

**Affiliations:** Institute of Nursing Science, University of Education Schwaebisch Gmuend, 73525 Schwaebisch Gmuend, Germany; simonehalder@gmx.de (S.H.);

**Keywords:** health literacy, nursing, nursing students, COVID

## Abstract

Health literacy can be defined as an individual’s competence to use knowledge and information to maintain and improve health. Research has shown the crucial importance of health literacy in everyday life. Nurses play an important role in fostering patients’ health literacy. But there is a lack in research on nurses’ health literacy and how it influences their work. Therefore, this study aims to determine nursing students’ health literacy and find out whether this group assessed that there was a change in their health literacy before and after the COVID-19 pandemic. In addition, this study aims to find out whether there is a relation between nursing students’ assessments of their health literacy and their assessments of whether and how they use strategies to foster their patients’ health literary in everyday work. We conducted a longitudinal study with two points of measurement, before the COVID-19 pandemic (N = 112) and after (N = 304). Nursing students filled out an online questionnaire using validated scales. To analyze the data, we used descriptive statistics, correlation analysis, and Welch’s *t*-test. The results show that before the COVID-19 pandemic, the nursing students assessed their health literacy as quite high, and after the COVID-19 pandemic, they found it difficult to access (*t* = 17.881; *p* < 0.001; Cohen’s d = 0.46), understand (*t* = 16.404; *p* < 0.001; Cohen’s d = 0.56), appraise (*t* = 15.429; *p* < 0.00; Cohen’s d = 0.47), and apply health-related information (*t* = 13.761; *p* < 0.001; Cohen’s d = 0.54). Implications of our study concern nurses’ vocational education and training in which nurses must learn about health literacy and strategies to foster their patients’ health literacy.

## 1. Introduction

The COVID-19 pandemic showed the crucial importance of health literacy in everyday life because people had to “acquire and apply health information and adapt their behavior at a fast pace” [1] (p. 2). Moreover, research has shown that poor health literacy is “an underestimated public health problem globally” [1] (p. 2).

Health literacy can be described as individuals’ abilities to “obtain and translate knowledge and information in order to maintain and improve health in a way that is appropriate to the individual and system contexts” [2] (p. 1). Research on health literacy has rapidly increased in recent years; in particular, the period between 1997 and 2007 saw a tenfold increase in studies in this area [3]. There is agreement among researchers [4] that there is a relationship between “low levels of health literacy and poor health outcomes” [5] (p. 102688). During the earliest years of health literacy research, the concept of health literacy was largely understood as a set of skills, although there was also much interest in developing measurements of health literacy. Later health literacy research “redirected the interest from an analysis of literacy as a set of purely technical coding and decoding skills to the examination of literacy (…) embedded in broader social goals and imperatives” [6] (p. 61). Current research in this area is interested in different forms of health literacy. For instance, many studies focus on digital health literacy [7] as an individual’s ability to use digital health resources [8], while other studies are interested in mental health literacy [9].

There is agreement among scholars that the COVID-19 pandemic first revealed the importance and need for health literacy to many people [10,11]. For the first time in their lives, many people found that they were dependent on obtaining valid information to carry out their daily activities. People had to find out what kinds of restrictions were imposed by their governments and how they could protect their health, for instance, by wearing masks. Consequently, people needed to find valid information and apply it in their daily lives. Indeed, studies have demonstrated that “better health literacy is associated with better attitudes towards preventive strategies against COVID-19” [12] (p. 1).

Healthcare workers and nurses play an important role in fostering people’s health literacy, including outside the COVID-19 pandemic [13]. While many studies emphasize the importance of nurses’ health literacy [14], there are three gaps in the research. First, little is known about nurses’ health literacy and how it influences their work, although it can be assumed that nurses with high personal health literacy are also interested in fostering their patients’ health literacy [15,16]. Second, few studies focus on nursing students and the health literacy of this group, and third, few studies have addressed nurses’ health literacy in regard to the COVID-19 pandemic. In particular, there is a lack of studies comparing nurses’ health literacy before and after the pandemic.

Therefore, this study focuses on nursing students’ health literacy. The aims are to determine nursing students’ health literacy, to find out whether this group assessed that there was a change in their health literacy before and after the COVID-19 pandemic, and whether nurses use strategies to foster their patients’ health literacy. The research questions of this study are as follows:

(1) How do nursing students assess their health literacy?
(2) Is there a relation between nursing students’ assessments of their health literacy and their assessments of whether and how they use strategies to foster their patients’ health literary?
(3) Is there a difference between nursing students’ assessments of their health literacy before and after COVID-19?

## 2. Theoretical Background

### 2.1. Definitions of Health Literacy

Health literacy research has used many different definitions and operationalizations of the construct. Regarding the definitions, Nutbeam differentiated three different approaches. First, health literacy can be regarded as “a risk factor” [17] (p. 2072). In this perspective, health literacy is used to describe a “set of individual literacy capacities that act as a mediating factor in health and clinical decision-making” [17] (p. 2073). One prominent definition emerging from this perspective was developed by the US National Academy of Medicine, which defined health literacy as “the degree to which individuals have the capacity to obtain, process and understand basic health information and services needed to make appropriate health decisions” [17] (p. 2073). The second approach sees “health literacy as (an) asset” [17] (p. 2074). This perspective on health literacy originally appeared in the field of public health and defines health literacy as “an asset to build, as an outcome to health education and communication that supports greater empowerment in health decision-making” [17] (p. 2074). A further definition that could fall under this approach was proposed by Urstad et al. [18] (p. 1), who pointed out that “health literacy is usually understood as cognitive and social skills that determine the motivation and ability to understand and use health information, and adequate health literacy is seen as a prerequisite for healthy behaviors”. This third perspective is broader than the others and does not focus only on possessing a set of competencies or skills. In this definition, health literacy could be understood as “the achievement of a level of knowledge, personal skills and confidence to take action to improve personal and community health by changing personal lifestyles and living conditions” [17] (p. 2074).

To show “the deeper meaning and purpose of health literacy for people’s life” [17] (p. 263), Nutbeam et al. [17] differentiated three different levels of health literacy: basic and functional health literacy, communicative and interactive health literacy, and critical literacy. Basic and functional health literacy means “having basic skills in reading and writing to be able to function effectively in everyday situations” that have a relation to health [17] (p. 263). Communicative and interactive health literacy, meanwhile, means higher competencies that allow individuals to obtain health information from different sources and apply it in useful ways. Finally, critical literacy can be described as “advanced cognitive skills which together with social skills can be applied to critically analyze information” [17] (p. 264).

The current study focuses on health literacy as a set of competencies and skills “to communicate and interact with healthcare providers as well as the ability to interpret and critically analyze health information” [18] (p. 5). Sørensen et al. [19] proposed this definition of health literacy and developed a model that describes different dimensions of the construct.

### 2.2. Model of Health Literacy

Based on a systematic review, Sørensen et al. [19] developed a conceptual model of health literacy “that captures the most comprehensive evidence-based dimensions of health literacy with its main antecedents and consequences” [19] (p. 1053). The same authors defined health literacy as “the knowledge, motivation and competences to access, understand, appraise and apply health information in order to make judgments and take decisions in everyday life concerning healthcare, disease prevention and health promotion to maintain or improve quality of life throughout the course of life” [19] (p. 1052).

The model shows four different key competencies that focus on the process of “accessing, understanding, appraising and applying health-related information” [19] (p. 8). The competence of accessing can be described as the “ability to seek, find and obtain health information”, while that of understanding can be understood as the “ability to comprehend the health information that is accessed” [19] (p. 9). The third competence, appraising, refers to the “ability to interpret, filter, judge and evaluate the health information that has been accessed,” and the fourth, which focuses on applying, is the “ability to communicate and use information to make a decision to maintain and improve health” [19] (p. 9).

### 2.3. Nurses’ Health Literacy and Health Literacy Strategies at Work

Research has revealed that nurses’ health literacy is related to improving patients’ health literacy [20,21,22]. Studies indicate that healthcare workers do have a positive attitude towards health literacy and are convinced that patients’ health literacy should be fostered through their work [23]. Nursing organizations in which nurses can effectively communicate with patients and “appropriately and adequately assess the health literacy of their patients” provide higher-quality healthcare delivery and improve patients’ health literacy [24]. Referring to Nutbeam et al.’s [17] levels of health literacy, it can be assumed that nurses need to have critical literacy, as this will enable them to both gain health-related skills and critically analyze information. Critical literacy can be described as “advanced cognitive skills which together with social skills can be applied to critically analyze information” [17] (p. 264). Nurses must analyze information to use it “in the assessment of patients’ clinical conditions and need to provide education and disease management based on patients’ ability to understand” and must therefore have “awareness, knowledge, skills, and attitudes” in health literacy [20] (p. 579).

Cafiero [22] focused on the health literacy strategies of nurses in practice settings. Health literacy strategies can be defined as the strategies used by nurses to communicate with and inform patients who have low health literacy or want to improve their health literacy [25]. Such strategies include, for example, using simple language and explaining medical and nursing-specific words as well as showing patients how to obtain and analyze information. Nurses’ health literacy strategies include nurses’ knowledge of health literacy, their experience of using health literacy strategies, and their intentions to use such strategies in their nursing work.

### 2.4. Health Literacy and the COVID-19 Pandemic

Research has shown that “applying critical health literacy has never been more needed than in these days when an infectious disease crisis arrives at a time of information excess and high expectations of controlling health” [26] (p. 1612). Patil et al. [27] showed in their study on health literacy during the COVID-19 pandemic that health literacy was related to an overall compliance with basic preventive practices, such as mask-wearing. Furthermore, they found that lower health literacy was related to the assumption that the response to the pandemic “was an overreaction” (p. 3301). Other studies, such as that by Okan et al. [28], showed that people with lower health literacy felt more confused about coronavirus information. Anselmann and Bohn [29] focused on nurses’ professional identities, anxiety, and coping strategies during the COVID-19 pandemic and found that nurses acted in various ways. Nurses who identified more with knowledge and skills were more satisfied with information about the crisis and felt lower anxiety than those who assessed themselves as possessing less knowledge and fewer skills.

Research indicates that the need for health literacy changed during the COVID-19 pandemic. As noted above, the fact that many people found obtaining valid health information to be important for their daily lives and health caused them to value health-related information more than they had done previously [30]. Therefore, it can be assumed that estimations of health literacy changed during the COVID-19 pandemic. The current study therefore aims to determine whether nursing students believe that their health literacy and health literacy strategies have changed since the COVID-19 pandemic.

## 3. Materials and Methods

We conducted a longitudinal study with two points of measurement, before (T0) and after (T1) the COVID-19 pandemic, and used unpaired sampling.

### 3.1. Data Collection and Sample

Data at T0 were collected at the beginning of 2020. We contacted six different vocational colleges in Germany and asked their principals to facilitate their students’ participation. We used a paper–pencil questionnaire, and 112 nursing students (N = 112) participated in our study.

13 (22%) participants were in their third semester of their vocational education and training (VET), while 16 participants (27.1%) were in the fourth, and 29 (45.5%) participants were in the final years of their VET. With regard to sex, 45 participants (75.9%) were female, and 42 participants (81.9%) were under the age of 24.

For T1, data were collected in November 2022. We contacted 84 vocational colleges in Germany and asked the principals to forward the link to our online questionnaire to their students. In total, 304 (N = 304) nursing students participated in our study of whom 241 participants (66.4%) were female. Moreover, 80 participants (22.0%) were in their first semester of VET, 42 participants (12.7%) were in their second, 108 participants (29.8%) were in their third, 8 participants (2.2%) were in their fourth, 40 participants (11.0%) were in their fifth, and 12 participants (3.3%) were in their final semesters. In terms of age, 92 participants (25.3%) were under 22.

Participation at both measurement points was voluntary. A statement at the beginning of the questionnaires informed the participants that the data collected would be anonymized. Participants were informed that their participation could not be traced back by either the researchers or the principals. Ethical approval was obtained from the ethics committee of the University of Education Schwäbisch Gmünd.

### 3.2. Instrument

At both measurement points, an identical questionnaire with validated scales was used. To measure health literacy, we used the health literacy scale (HLS-EU-Q) developed by Sørensen et al. [31]. This scale has four subscales regarding accessing, understanding, appraising, and applying health-relevant information. The scale was combined with a 4-point Likert scale (where 1 = very difficult and 4 = very easy). An item example for the subscale is “On a scale from very easy to very difficult, how easy would you say it is to find out where to get professional help when you are ill”. To measures nursing students’ strategies to improve their patients’ health literacy, we used the Health Literacy Strategies Behavioral Intention Questionnaire developed by Cafiero et al. [32]. The scale was combined with a 5-point Likert scale ranging from 1 = never to 5 = very often. An item example is “How often did you use a health literacy screening tool to assess health literacy?” We also collected sociodemographic data on age, gender, and semester of the VET.

### 3.3. Analysis

To analyze the data generated, we first analyzed reliability and estimated the Cronbach’s alpha of all scales. We used descriptive statistics to show means and standard deviations. To determine whether there was a relation between nursing students’ health literacy and their strategies to foster patients’ health literacy, we used correlation analysis. To analyze whether a difference existed between the two groups and their estimated health literacy, we used Welch’s *t*-test in SPSS [33].

## 4. Results

For answering research question 1 on nurses’ estimation of their own health literacy, we used descriptive statistics. Correlation analysis was used to find out whether there was a relation between nurses’ self-perceived health literacy and their use of strategies to foster health literacy in their patients. To answer research question 3, we tested for differences by using ANOVA.

### 4.1. Descriptive Statistics and Cronbach’s Alpha

The results at T0 show that nursing students felt competent in health literacy. Before the COVID-19 pandemic, nursing students thought it was very easy to understand health information. Regarding their strategies to promote patients’ health literacy, nursing students did not use strategies very often. Table 1 presents the results.

The results of T1 show that nursing students think that it is easy to understand (M = 1.93; SD = 0.46) health-related information. Appraising health information is estimated as difficult by them (M = 2.30; SD = 0.50). Nursing students indicate that they use health literacy strategies not very often (M = 2.73; SD = 0.59). In T1, nursing students indicate all aspects of health literacy as difficult. In comparison, understanding health-related information (M = 3.24; SD = 0.57) is estimated as not so difficult than the other aspects. The results for T1 (see Table 2) show that the nursing students assessed their health literacy as lower than at T0. The nursing students assessed appraising information as very difficult. Regarding health literacy strategies, the nursing students thought that they applied strategies very often (M = 3.44; SD = 0.77).

### 4.2. Correlation Analysis

To determine whether a relation existed between the different dimensions of health literacy and nursing students’ strategies to foster their patients’ health literacy, we used correlation analysis. Our results for T0 showed that there was no significant correlation between Accessing and nurses’ strategies to foster patient’s health literacy (r = 0.10; *p* = 0.43) and no significant correlation between Understanding and nurses’ strategies to foster patient’s health literacy (r = −0.09; *p* = 0.47). There were no significant correlations between Appraising and nurses’ strategies to foster patient’s health literacy (r = 0.12; *p* = 0.36) and no significant correlation between Applying and nurses’ strategies to foster patient’s health literacy (r = 0.17; *p* = 0.19). The results for the data collected at T1 showed that there was a significant positive relation between the dimensions Accessing and nurses’ strategies to foster patient’s health literacy (r = 0.25; *p* = 0.001), Understanding and nurses’ strategies to foster patient’s health literacy (r = 0.25; *p* = 0.001), Appraising and nurses’ strategies to foster patient’s health literacy (r = 0.23; *p* = 0.001), and Applying and nurses’ strategies to foster patient’s health literacy (r = 0.24; *p* = 0.001).

### 4.3. Testing for Differences

To determine whether there was a significant difference between nursing students’ estimations of their health literacy before and after the COVID-19 pandemic, we used a one-way analysis of variance (ANOVA). The results showed that there were significant differences for all dimensions of health literacy. In detail, there was a significant difference for the Accessing (*t* = 17.881; *p* < 0.001; Cohen’s d = 0.46), Understanding (*t* = 16.404; *p* < 0.001; Cohen’s d = 0.56), Appraising (*t* = 15.429; *p* < 0.00; Cohen’s d = 0.47), and Applying (*t* = 13.761; *p* < 0.001; Cohen’s d = 0.54) dimensions. Furthermore, there was a significant difference regarding nurses’ strategies to foster patient’s health literacy (*t* = 6.709; *p* < 0.001; Cohen’s d = 0.74). As a post hoc test, we used Welch’s *t*-test and the Brown–Forsythe test. The results also showed significant differences for all variables. Table 3 presents all the results.

## 5. Discussion

The results of our study indicate that the nursing students’ assessments of their own health literacy were different at the two measurement points, that is, before and after the COVID-19 pandemic. While before the COVID-19 pandemic, the nursing students assessed their health literacy as quite high, after the COVID-19 pandemic, they found it difficult to access, understand, appraise, and apply health-related information. Regarding their health literacy strategies at work, we found that before the COVID-19 pandemic, nursing students assessed their engagement in fostering their patients’ health literacy as quite low, and after the COVID-19 pandemic, nursing students were interested in promoting their patients’ health literacy.

Studies on the health literacy of nursing students during the COVID-19 pandemic have mostly focused on the role of health literacy in, for instance, increasing health-related behavior or reducing anxiety [34]. Furthermore, although we found studies that examined COVID-19-related health literacy [28], we could not find another study focusing on two different points of measurement, namely, before and after the COVID-19 pandemic.

Our results could be explained by experiential learning theories, which define learning as “a process whereby knowledge is created through the transformation of experiences” and point out that ideas and estimations are not “fixed and immutable elements of thought but are formed and reformed through experience” [35] (pp. 26–38). Under such theories, learning describes facing challenges that cannot be solved with existing strategies. Developing new strategies and knowledge enables challenges to be met; consequently, “learning is the major process of human adaption” [35] (p. 32). Experiences do not automatically change an individual’s knowledge or attitude. Rather, learning is a “transactional process” [34] (p. 36) in which individuals must reconcile a situation and related experiences with their individual needs to control that situation and make sense of the new experiences.

The situations people experienced during the COVID-19 pandemic could be regarded as so new that they could not be automatically handled with existing strategies and knowledge and thus resulted in learning and the creation of new knowledge. In this case, the pandemic situation changed the meaning and importance of health literacy. While before COVID-19, nursing students believed that they could obtain health-related information, during the pandemic, they experienced a flood of information, disinformation, and helpful information. In this situation, their abilities to find helpful information were challenged in ways they had never experienced. They had to gain new knowledge to, for instance, be able to identify “fake information”. In this process, the meaning and importance of the ability changed, as did the situation. The amount of fake information rose enormously during the pandemic [36]; therefore, it became much more complex and difficult to find valid information. This increased complexity may have also changed their assessment of their health literacy. An alternative explanation might be based on the Dunning–Kruger effect, which explains individuals’ overestimation of their knowledge and skills by not being able to realize their lack thereof [37].

The limitations of our study concern the different samples and the sample sizes at the two points of measurement. In T0, we had 112 participants, and in T1, 304 nursing students participated in our study. To analyze the data, we used Welch’s *t*-test, which enables a comparison of samples of different sizes.

## 6. Conclusions

The implications of our study concern nurses’ VET. The importance and meaning of health literacy were demonstrated by the COVID-19 pandemic, and nurses must learn about health literacy and strategies to foster their patients’ health literacy. Therefore, we assume that health literacy must be a part of their VET. Our findings show the relation between nursing students’ individual health literacy and their engagement in promoting their patients’ health literary.

## Figures and Tables

**Table 1:** Descriptive statistics and Cronbach’s alpha: T0. (* 4-point Likert scale: 1 = very easy to 4 = very difficult; ** 5-point Likert scale: 1 = never to 5 = always; M = mean; SD = standard deviation.)

Scale (Number of Items)	Example	Alpha	M	SD
Health Literacy *	On a scale from very easy to very difficult, how easy would you say it is to:			
Accessing (13)	…find information about symptoms of illnesses that concern you?	0.78	2.14	0.37
Understanding (11)	…understand what your doctor says to you?	0.80	1.93	0.46
Appraising (12)	…judge how information from your doctor applies to you?	0.85	2.30	0.50
Applying (11)	…call an ambulance in an emergency?	0.72	2.19	0.37
Health Literacy Strategies ** (6)	Use of health literacy strategies with patients would help patients stay healthy.	0.77	2.73	0.59

**Table 2:** Descriptive statistics and Cronbach’s alpha: T1. (* 4-point Likert scale: 1 = very easy to 4 = very difficult; ** 5-point Likert scale: 1 = never to 5 = always; M = mean; SD = standard deviation.)

Scale (Number of Items)	Example	Alpha	M	SD
Health Literacy *	On a scale from very easy to very difficult, how easy would you say it is to:			
Accessing (13)	…find information on treatments of illnesses that concern you?	0.83	3.30	0.47
Understanding (11)	…understand the leaflets that come with your medicine?	0.80	3.24	0.57
Appraising (12)	…judge the advantages and disadvantages of different treatment options?	0.84	3.34	0.46
Applying (11)	…find information about vaccinations and health screenings that you should have?	0.81	3.24	0.56
Health Literacy Strategies ** (6)	Improved patient understanding will improve patient outcomes.	0.83	3.44	0.77

**Table 3:** Means and *t*-test (* 4-point Likert scale: 1 = very easy to 4 = very difficult; ** 5-point Likert scale: 1 = never to 5 = always; M = mean; SD = standard deviation.)

Scale	Study 2		Study 1		*t*-Test	Cohen’s d
	M	SD	M	SD		
Health Literacy *						
Accessing	2.14	0.37	3.30	0.47	*t* = 17.881; *p* < 0.001	d = 0.46
Understanding	1.93	0.46	3.24	0.57	*t* = 16.404; *p* < 0.001;	d = 0.56
Appraising	2.30	0.50	3.34	0.46	*t* = 15.429; *p* < 0.00	d = 0.47
Applying	2.19	0	3.24	0.56	*t* = 13.761; *p* < 0.001;	d = 0.54
Health Literacy Strategies **	2.73	0.59	3.44	0.77	*t* = 6.709; *p* < 0.001	d = 0.74

## Data Availability

The datasets presented in this article are not readily available, to ensure the confidentially and anonymity of participants.
